# Precursor Chemistry Enables the Surface Ligand Control of PbS Quantum Dots for Efficient Photovoltaics

**DOI:** 10.1002/advs.202204655

**Published:** 2022-11-16

**Authors:** Chao Wang, Yinglin Wang, Yuwen Jia, Hai Wang, Xiaofei Li, Shuai Liu, Xinlu Liu, Hongbo Zhu, Haiyu Wang, Yichun Liu, Xintong Zhang

**Affiliations:** ^1^ Key Laboratory of UV‐Emitting Materials and Technology of Chinese Ministry of Education Northeast Normal University Changchun 130024 China; ^2^ State Key Laboratory on Integrated Optoelectronics College of Electronic Science and Engineering Jilin University Changchun 130012 China

**Keywords:** hydroxyl ligand, PbS colloidal quantum dots, precursor, trap state, water

## Abstract

The surface ligand environment plays a dominant role in determining the physicochemical, optical, and electronic properties of colloidal quantum dots (CQDs). Specifically, the ligand‐related electronic traps are the main reason for the carrier nonradiative recombination and the energetic losses in colloidal quantum dot solar cells (CQDSCs), which are usually solved with numerous advanced ligand exchange reactions. However, the synthesis process, as the essential initial step to control the surface ligand environment of CQDs, has lagged behind these post‐synthesis ligand exchange reactions. The current PbS CQDs synthesis tactic generally uses lead oxide (PbO) as lead precursor, and thus suffers from the water byproducts issue increasing the surface‐hydroxyl ligands and aggravating trap‐induced recombination in the PbS CQDSCs. Herein, an organic‐Pb precursor, lead (II) acetylacetonate (Pb(acac)_2_), is used instead of a PbO precursor to avoid the adverse impact of water byproducts. Consequently, the Pb(acac)_2_ precursor successfully optimizes the surface ligands of PbS CQDs by reducing the hydroxyl ligands and increasing the iodine ligands with trap‐passivation ability. Finally, the Pb(acac)_2_‐based CQDSCs possess remarkably reduced trap states and suppressed nonradiative recombination, generating a certified record *V*_oc_ of 0.652 V and a champion power conversion efficiency (PCE) of 11.48% with long‐term stability in planar heterojunction‐structure CQDSCs.

## Graphical abstract

Compared with the traditional PbO precursor synthesis process of PbS CQDs, lead acetylacetonate (Pb(acac)_2_) precursor can remarkably suppress the number of OH ligands on the surface of CQD, which is beneficial to increasing the iodine ligands with the trap‐passivation ability and improving the efficiency of PbS CQD solar cells.

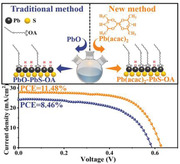

## 1 Introduction

With size‐tunable and shape‐tunable optical/electronic properties, as well as low‐cost, facile solution processability, semiconductor colloidal quantum dots (CQDs) are outstanding material candidates for both complex optoelectronic mechanisms and promising applications,^[^ ^1^, ^2^, ^3^ ^]^ including photodetectors,^[^ ^4^, ^5^, ^6^ ^]^ light‐emitting diodes,^[^ ^7^, ^8^ ^]^ photocatalysis,^[^ ^9^ ^]^ biological imaging,^[^ ^10^ ^]^ and photovoltaic cells.^[^ ^11^, ^12^, ^13^, ^14^, ^15^ ^]^ In particular, lead chalcogenide (PbX, X = S, Se) CQDs are promising wide‐spectrum light‐harvesting materials for solar cells, which are expected to break the Shockley−Queisser efficiency limit with the multiple exciton generation effect.^[^ ^16^ ^]^ The physicochemical, optical, and electronic properties of CQDs are significantly determined by their surface ligand environment.^[^ ^17^ ^]^ In general, the long‐chain ligands introduced by the synthesis process preclude agglomeration and ensure the monodispersity of CQDs due to the steric effect.^[^ ^12^ ^]^ Small‐sized organic ligands or inorganic ions are indispensable for enhancing the inter‐dot electronic coupling during the device fabrication process.^[^ ^18^, ^19^, ^20^ ^]^ More importantly, surface ligand modulation has become an essential process to passivate CQD surface defects for the reduction of nonradiative recombination in efficient CQDSCs. This is usually realized through a ligand exchange process performed after the synthesis step of CQDs. Various optimizations of the ligand exchange process, such as solvent engineering,^[^ ^21^, ^22^ ^]^ matrix engineering,^[^ ^23^, ^24^ ^]^ hybrid passivation,^[^ ^25^ ^]^ and stepwise passivation,^[^ ^26^ ^]^ have enhanced the efficiency of PbS CQDSCs by up to 14%.^[^ ^27^ ^]^

However, the surface ligand environment of CQDs is initially formed during the synthesis process. Unfortunately, the development of the PbS CQD synthesis method for surface ligand modulation has lagged greatly behind that of post‐synthesis ligand exchange reactions, resulting in several unsolved surface issues blocking the efficiency improvement of PbS CQDSCs. Typically, PbS CQDs are produced with the Hines synthesis method.^[^ ^28^ ^]^ This begins with a reaction between PbO precursor and oleic acid, generating lead oleate (Pb(OA)_2_) intermediates and water byproducts. These water byproducts cannot be entirely removed from the reaction system and they introduce surface hydroxyl (OH) ligands on Pb‐terminated polar (111) facets during the nucleation and growth process of CQDs.^[^ ^29^ ^]^ OH ligands have been reported as the main reason for the surface defects of PbS CQDs and are difficult to remove from CQD surfaces with postsynthetic ligand exchange due to their high binding energy.^[^ ^30^, ^31^, ^32^ ^]^ Additionally, they can trigger the aggregation of CQDs through the strong hydrogen bond with water in the air.^[^ ^33^ ^]^ These effects of OH ligands exacerbate the nonradiative carrier recombination of PbS CQDs and thus result in the serious energetic loss of CQDSCs.^[^ ^16^ ^]^ Therefore, an alternative synthesis method is imperative to eliminate water byproducts and further suppress the surface OH of PbS CQDs, achieving the surface ligand control from the root for advanced CQDSCs.

In this research, a metal‐organic Pb precursor, Pb(acac)_2_, was utilized for the first time in the hot‐injection synthesis of PbS CQDs for high‐efficiency CQDSCs. In contrast to the generally used PbO precursor, Pb(acac)_2_ reacts with oleic acid through the ligand exchange process and generates Pb(OA)_2_ intermediates with the acetylacetone byproducts. The acetylacetone has good solubility in organic solvents, making it easy to remove from the reaction system. Pb(acac)_2_ avoids the water byproducts without affecting the nucleation and growth of PbS CQDs, and thus successfully solves the aforementioned issues of water‐generated hydroxyl ligands during the synthesis process of CQDs. Compared with the CQDs formed by PbO, those from a Pb(acac)_2_ precursor exhibit higher iodine ligands content after ligand exchange, ensuring the reduction of the band‐tail trap states and the suppression of the carrier recombination. As a result, Pb(acac)_2_ has generated a certified record *V*_oc_ of 0.652 V in the planar heterojunction CQDSCs and successfully achieved the champion PCE of 11.48%. Our work confirmed Pb(acac)_2_ as an ideal Pb precursor to optimize the surface ligand environment of PbS CQDs and reduce trap‐related energetic loss for high‐performance photovoltaics.

## 2 Results and Discussion

**Figure 1:**
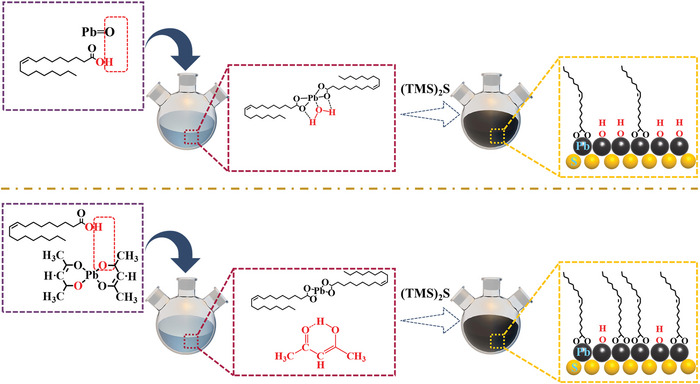
Schematic representation of PbS CQD synthesis process using lead (II) oxide and lead (II) acetylacetonate precursors.

In the conventional PbS CQD synthesis method (**Figure** **1**), PbO precursor is first decomposed by hot oleic acid, which forms lead oleate (Pb(OA)_2_) intermediate (called PbO‐Pb(OA)_2_) and water byproducts. Previous literatures reported that these water byproducts were difficult to remove from the reaction system due to their strong hydrogen bond with carboxylate‐Pb species, and finally generated surface OH ligands during the nucleation and growth progress of CQDs.^[^ ^34^ ^]^ In contrast, we dissolved Pb(acac)_2_ precursor in oleic acid to generate Pb(OA)_2_ intermediate (called Pb(acac)_2_‐Pb(OA)_2_) and acetylacetone byproducts, as shown in Figure 1. Acetylacetone is very soluble in an organic solvent and has difficulty with carboxylate‐Pb species because of its steric hindrance. As a result, the acetylacetone byproducts were expected to be eliminated during the rinsing process of the PbS CQDs. In addition, the FTIR results of the precursor solution (reaction solution of oleic acid and Pb(acac)_2_ in octadecene) shown in Figure S1 (Supporting Information) exhibited characteristic peaks in the enol‐structure acetylacetone at 1620, 1240, and 953 cm^−1^ representing the C = O stretching, C−O stretching and O−H∙∙∙O stretching, respectively, while the characteristic peak of keto‐structure acetylacetone (C = O stretching at ≈1730 cm^−1^) was barely observed. Thus, the acetylacetone byproducts mainly existed as an enol form in the reaction system, probably due to the stable six‐membered ring‐like structure of the enol structure (Figure S2, Supporting Information).

**Figure 2:**
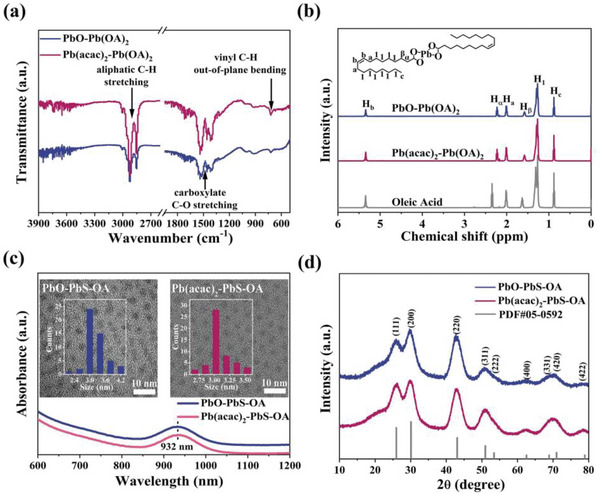
Characterization of the Pb(OA)_2_ intermediate and synthesized PbS CQD capped with OA ligands. a) FTIR spectra of PbO‐Pb(OA)_2_ and Pb(acac)_2_‐Pb(OA)_2_, b) ^1^H NMR spectra of PbO‐Pb(OA)_2_ and Pb(acac)_2_‐Pb(OA)_2_, c) Absorption spectra of PbO‐PbS‐OA and Pb(acac)_2_‐PbS‐OA. The inset shows the TEM images of the PbS‐OA and size distribution. d) X‐ray diffraction patterns of the PbO‐PbS‐OA and Pb(acac)_2_‐PbS‐OA.

The Fourier transform infrared spectroscopy (FTIR) result of the Pb(acac)_2_‐Pb(OA)_2_ and PbO‐Pb(OA)_2_ intermediates presented an inconspicuous difference, exhibiting characteristic peaks of the C−H stretching of the aliphatic chains at ≈2910 cm^−1^ and the C−O stretching of the carboxylate group at ≈1510 cm^−1^ (**Figure** **2**a). In addition, as shown in Figure 2b, the nuclear magnetic resonance (NMR) spectra of PbO‐Pb(OA)_2_ and Pb(acac)_2_‐Pb(OA)_2_ in CDCl_3_ showed oleyl moieties at the chemical shift of 5.34 ppm, which were in line with pure oleic acid. Additionally, the NMR peaks of the protons in the alpha and beta positions (H_*α*_ and H_*β*_) of the Pb(OA)_2_ (PbO‐Pb(OA)_2_ and Pb(acac)_2_‐Pb(OA)_2_) shifted to a higher field in comparison with oleic acid, which suggested the coordination of the Pb atom with oleate.^[^ ^35^ ^]^ This verified that we successfully synthesized Pb(OA)_2_ intermediate with Pb(acac)_2_ despite the generation of different byproducts. It is worth noting that we did not observe the peak of water in the NMR spectra of PbO‐Pb(OA)_2_, which was likely because the signal of the hydrate in the dimer was too weak to be detected.

These two Pb(OA)_2_ intermediates further reacted with the injected hexamethyldisilathiane (TMS_2_S) sulfur precursor and finally generated oleate‐capped PbS CQDs, denoted as PbO‐PbS‐OA CQDs and Pb(acac)_2_‐PbS‐OA CQDs according to the Pb precursor. The absorption spectra (Figure 2c) suggested that the PbO‐PbS‐OA and Pb(acac)_2_‐PbS‐OA CQD solutions exhibited the same first exciton absorption peaks at 932 nm. We calculated the diameters (*d*) of both PbS‐OA CQDs to be around 3 nm using the equation *E*_0_ = 0.41 + (0.0252*d* ^2^ + 0.283*d*)^−1^, where *E*_0_ is the optical bandgap of the PbS CQDs obtained from the first exciton absorption peaks.^[^ ^36^ ^]^ The size distribution of the two samples measured with the transmission electron microscopy (TEM) images (inset of Figure 2c) provided good agreement with the calculated *d* from the absorption spectra. The X‐ray diffraction (XRD) peaks of PbO‐PbS‐OA and Pb(acac)_2_‐PbS‐OA shown in Figure 2d were almost identical. These results demonstrated that the replacement of the PbO precursor by Pb(acac)_2_ induced an unobservable change in the inorganic core of the OA‐capped PbS CQDs. In addition, in the TEM images shown in the inset of Figure 2c and Figure S3 (Supporting Information), we observed a superior monodispersity of Pb(acac)_2_‐PbS‐OA in contrast to the conspicuous fusion of PbO‐PbS‐OA.

We further investigated the precursor‐induced surface ligand difference between these two PbS‐OA CQDs. The FTIR spectrum of PbO‐PbS‐OA in Figure S4a (Supporting Information) displayed significant broad peaks around 3450 cm^−1^, which were attributed to the Pb−OH stretching vibration.^[^ ^35^ ^]^ In contrast, the Pb−OH peak intensity of the Pb(acac)_2_‐PbS‐OA was largely reduced. X‐ray photoelectron spectroscopy (XPS) was applied to gain insight into the change of the OH ligands in the PbS‐OA CQDs (Figures S4b,c, Supporting Information). The O1s peak of PbS‐OA was deconvoluted into three species. The species at 531.0 eV could be assigned to the hydroxyl ligand (Pb−OH) binding to PbS CQDs, the peak emerging at 532.1 eV (C−O) could be attributed to oleate ligands or CO_2_ and the peak at 529.6 eV was Pb−O, as summarized in Table S1 (Supporting Information). The peak intensity of the Pb−OH for the Pb(acac)_2_‐PbS‐OA prominently decreased in comparison with that of the PbO‐PbS‐OA. Based on the results of FTIR and XPS, we confirmed that the surface OH ligands of the PbS‐OA CQDs could be effectively reduced by employing Pb(acac)_2_ as Pb precursor. In addition, the absorbance and NMR measurements showed that the density (*∑*_OA_) of the oleate ligand (per unit area) on the surface of the CQD prepared with Pb(acac)_2_ precursor (6.80 nm^−2^) was higher than that of the PbO based counterparts (5.04 nm^−2^), as shown in Figure S5 and Table S2 (Supporting Information). This implied that the Pb(acac)_2_‐induced reduction of the OH ligands could provide additional binding sites for OA ligands on the surface of the PbS CQDs.

**Figure 3:**
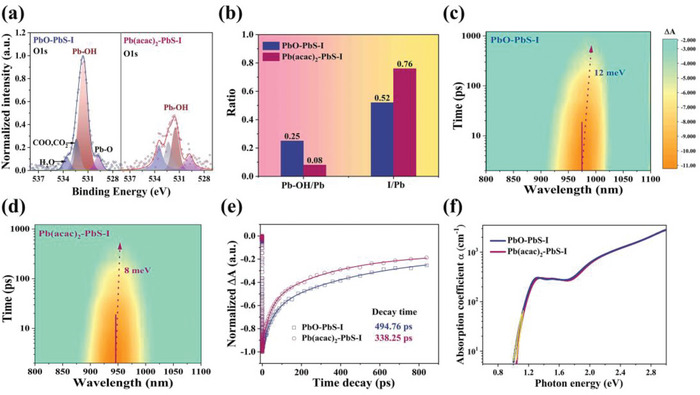
Characterizations of PbS‐I films. a) XPS spectra of PbS‐I films in O1s of PbO‐PbS‐I and Pb(acac)_2_‐PbS‐I. b) The ratio of Pb−OH/Pb and I/Pb. Spectro‐temporal transient absorption (TA) map for c) PbO‐PbS‐I film and d) Pb(acac)_2_‐PbS‐I film. e) Decay curve from TA panel. f) Urbach energies for the two films.

The long‐chain ligands of the PbS‐OA had to be exchanged by small‐sized iodine ligands for the strong inter‐dot electronic coupling in the photovoltaic device, and it was of great importance to study whether the effect of Pb(acac)_2_ on suppressing the OH ligands that still remained after the ligand exchange process. Thus, we probed the surface ligands of the iodine‐capped CQDs, which were called PbO‐PbS‐I and Pb(acac)_2_‐PbS‐I according to the precursors. Similar to the PbO‐PbS‐OA, the PbO‐PbS‐I obtained with the ligand exchange still displayed obvious Pb−OH signals (531.5 eV) in the O1s XPS spectra (**Figure** **3**a). This indicated that the OH ligands introduced in the synthesis step were difficult to replace with the iodine ligands during the ligand exchange process, even though the iodine species was one of the promising ligands for the surface passivation of CQDs. By contrast, a considerably reduced area of the Pb−OH peak was observed in the Pb(acac)_2_‐PbS‐I, which could be confirmed by the ratios of Pb−OH/Pb (0.25 for PbO‐PbS‐I vs 0.08 for Pb(acac)_2_‐PbS‐I), as shown in Figure 3b, Table S3 and Table S4 (Supporting Information). Furthermore, we found that the Pb(acac)_2_‐PbS‐I film had a larger I/Pb ratio (0.76) than the PbO‐PbS‐I film (0.52) (Figure 3b, Figure S6a,b, Supporting Information). This indicated that more iodine ligands were bonded to the surface of the Pb(acac)_2_‐PbS‐I CQDs after the ligand exchange. The decrease of the OH ligands and/or the increase of the oleate ligands on the Pb(acac)_2_‐PbS‐OA CQD surface could provide more reactive surface sites to attach iodine ligands during the ligand exchange process. Consequently, the Pb(acac)_2_ precursor successfully modulated the surface ligand environment of the PbS‐I CQDs, reducing the OH ligands and increasing the iodine ligands.

As aforementioned, the surface ligand environment affects the surface defects of CQDs and changes the trap‐related nonradiative and/or radiative carrier recombination.^[^ ^37^ ^]^ Thus, we performed systematical optical measurements to explore the influence of the Pb(acac)_2_ precursor on the surface defects of the PbS‐OA CQDs. The steady photoluminescence peak of Pb(acac)_2_‐PbS‐OA showed a ≈10‐nm blueshift compared to the PbO‐PbS‐OA (Figure S7a, Supporting Information). Because the same first exciton absorption peaks existed two CQDs, we could confirm that the Pb(acac)_2_‐PbS‐OA had a smaller Stokes shift. This verified that the Pb(acac)_2_ precursor successfully suppressed the nonradiative carrier recombination of the PbS‐OA CQDs, which could also be further evidenced by the longer transient PL lifetime of the Pb(acac)_2_‐PbS‐OA than that of the PbO‐PbS‐OA (2.65 µs vs 2.20 µs, Figure S7b, Supporting Information). The OH ligands were considered to be an important source of surface defects for the PbS CQDs and to induce the fusion of adjacent PbS CQDs in the ambient condition.^[^ ^33^ ^]^ These adverse effects of OH ligands led to band‐tail trap states to aggravate the carrier nonradiative recombination of PbS CQDs. Thus, the monodispersity observed in the TEM image of the Pb(acac)_2_‐PbS‐OA (Figure 2c) and the suppressed nonradiative carrier recombination of the Pb(acac)_2_‐PbS‐OA were both ascribed to the effect of the Pb(acac)_2_‐precursor on decreasing the OH ligands and passivating the trap states.

As mentioned above, the high‐performance CQDSCs required the I‐capped PbS‐CQDs obtained with ligand exchange, so we studied the photophysical behaviors of two PbS‐I CQDs in detail. The PL peak of the Pb(acac)_2_‐PbS‐I in DMF solution showed a 12‐nm blueshift in comparison with that of the PbO‐PbS‐I (Figure S7c, Supporting Information), whose full width at half maximum (130 meV) is much higher than the that of Pb(acac)_2_‐PbS‐I (96 meV). In addition, as shown in Figure S7d (Supporting Information), the Pb(acac)_2_‐PbS‐I solution (2.88 µs) yielded a longer PL lifetime than the PbO‐PbS‐I (2.39 µs). Both the steady‐state and transient PL suggested that the Pb(acac)_2_‐precursor could also reduce the band‐tail states and attenuate the nonradiative recombination of the PbS‐I CQDs. Furthermore, the transient absorption (TA) spectroscopy panels displayed in Figure 3c,d showed a redshift of 8 meV for the Pb(acac)_2_‐PbS‐I film in the transient bleaching peak, while the PbO‐PbS‐I exhibited a 12 meV redshift, which illustrated the reduced energy funnel and lower band tail states in the Pb(acac)_2_‐PbS‐I film.

As shown in Figure 3e, the Pb(acac)_2_‐PbS‐I film exhibited a faster decay time of 338.25 ps compared to the 494.76 ps decay time of the PbO‐PbS‐I film, suggesting a faster charge‐transfer rate and stronger electronic coupling between dots in the Pb(acac)_2_‐PbS‐I film.^[^ ^38^ ^]^ Simultaneously, the Urbach energy (*E*_u_) was utilized to evaluate the energetic disorder in the PbS‐I CQDs films. As shown in Figure 3f, the *E*_u_ was determined to be 39.2 meV for the Pb(acac)_2_‐PbS‐I films. This was over 20% less than that of the PbO‐PbS‐I films (50.1 meV). The smaller *E*_u_ value indicated fewer band tail states in the Pb(acac)_2_‐PbS‐I film. This result, which was consistent with the results of the TA spectroscopy, correlated to a flatter energy landscape and better trap passivation.^[^ ^39^, ^40^ ^]^ Therefore, the Pb(acac)_2_ precursor also resulted in the enhanced passivation of the surface defects for the PbS‐I CQDs, similar to that observed for the PbS‐OA CQDs, which was related to the amount of variation of the OH and I ligands obtained from the XPS measurement.

In addition, the influence of this precursor‐induced ligand variation on the electrical properties of the PbS‐I CQD films was investigated using the space charge limited current (SCLC) measurements of the electron‐only devices (FTO/PbS‐I/Al). As can be seen in Figure S8 (Supporting Information), the trap‐filled limit voltages (*V*_TFL_) were 0.48 V for the PbO‐PbS‐I and 0.20 V for the Pb(acac)_2_‐PbS‐I. Therefore, the electron trap densities (*N*_trap_) of the PbO‐PbS‐PbI_2_ and the Pb(acac)_2_‐PbS‐PbI_2_ were calculated to be 3.9 × 10^15^ and 1.7 × 10^15^ cm^−3^, respectively. In addition, the mobility in the Pb(acac)_2_‐PbS‐I CQD film was evaluated to be 1.8 × 10^−3^ cm^2^ V^−1^ s^−1^, which was much higher than that of 9.2 × 10^−4^ cm^2^ V^−1^ s^−1^ for the PbO‐PbS‐I. The decreased trap density and the enhanced carrier mobility of the Pb(acac)_2_‐PbS‐I, obtained from the SCLC measurements, coincided well with the suppression of the nonradiative carrier recombination and the enhanced carrier transport observed from the aforementioned photophysical measurements.

**Figure 4:**
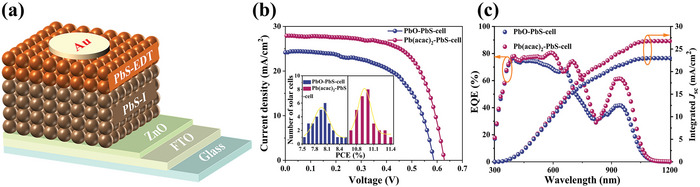
PbS device structure and performance plots. a) Schematic diagram of device construction in this work. b) Current density–voltage (*J*−*V*) characteristics with power conversion efficiency (PCE) distributions of both the PbO and Pb(acac)_2_‐based devices. c) EQE and integrated current.

The advantage of the Pb(acac)_2_ precursor in improving the efficiency of photovoltaic cells was confirmed using a general device structure with ZnO film as an electron extraction layer, PbS‐I as a light absorber layer, and 1,2‐ethanedithiol (EDT)‐treated PbS CQD as the p‐type hole extraction layer (**Figure** **4**a). The current density−voltage (*J*−*V*) characteristic curves (Figure 4b tested under AM1.5 G 100 mW cm^−2^ illumination) indicated that the solar cell obtained from the PbO precursor (PbO‐PbS‐cell) achieved a PCE of 8.26% with a *V*_oc_ of 0.585 V, a *J*_sc_ of 24.26 mA cm^−2^ and a fill factor (FF) of 58%. In contrast, the solar cell from the Pb(acac)_2_ precursor (Pb(acac)_2_‐PbS‐cell) generated a superior PCE of 11.48%, with a *V*_oc_ of 0.63 V, a *J*_sc_ of 27.93 mA cm^−2^ and a FF of 65%. The average parameters of the solar cells from 30 devices (**Table** **1**) confirmed a prominent improvement in the performance of the Pb(acac)_2_‐PbS‐cell. The PCE histograms (inset of Figure 4b) of the control and target devices showed reproducible efficiency. The external quantum efficiency (EQE) of the Pb(acac)_2_‐PbS‐cell exhibited a striking enhancement compared with that of the PbO‐PbS‐cell across the entire light‐response region (Figure 4c). *J*_sc_ integrated from the EQE spectra was in good agreement with the *J−V* results. Particularly, the Pb(acac)_2_ induced an EQE increase of about 20% at the first exciton absorption peak (930 nm), which was attributed to the flatter energy landscape and enhanced charge collection (*η*) in the Pb(acac)_2_‐PbS‐cell (Figure S9a, Supporting Information).

**Table 1:** Summary of photovoltaic parameters of PbO‐PbS‐cell and Pb(acac)_2_‐PbS‐cell devices based on 30 different solar cells

Device type	*V*_oc_ [V]	*J*_sc_ [mA cm^−2^]	FF [%]	PCE [%]	*R*_s_ [Ω cm^−2^]^a)^	*R*_sh_ [kΩ cm^−2^]^b)^
PbO	0.58 ± 0.02	24.25 ± 0.3	0.58 ± 0.02	8.0 ± 0.5	3.5 ± 0.1	0.46 ± 0.09
Champion device	0.585	24.44	0.59	8.46	3.6	0.51
Pb(acac)_2_	0.63 ± 0.02	27.84 ± 0.3	0.67 ± 0.02	11.1 ± 0.3	2.8 ± 0.1	1.26 ± 0.05
Champion device	0.630	27.93	0.65	11.48	2.8	1.29

^a)^ Series resistance (*R*_s_);

^b)^ Shunt resistance (*R*_sh_).

**Figure 5:**
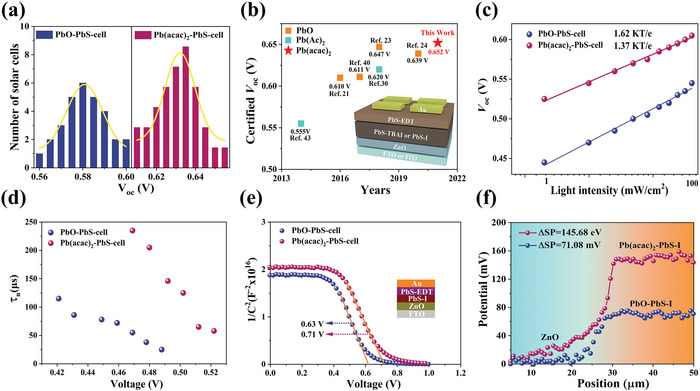
Device characterization of the PbS CQDs. a) *V*_oc_ distributions of PbO and Pb(acac)_2_ solar cells. b) Certified AM 1.5 *V*_oc_ of PbS CQDSCs fabricated with various Pb precursors. c) Light‐intensity dependence of *V*_oc_. d) Electron lifetimes of two devices for different bias voltages. e) Capacitance−voltage (*C*−*V*) curves. f) Surface potential profiles between the ZnO and the PbS–I film interface based on different Pb precursors.

We noticed that the Pb(acac)_2_‐PbS‐cells possessed a significantly higher *V*_oc_ than the PbO‐PbS‐cells. The *V*_oc_ distribution collected from 30 cells further confirmed this effect of the Pb(acac)_2_ precursor on improving *V*_oc_ (**Figure** **5**a). Encouragingly, we achieved an exceptional *V*_oc_ of 0.652 V from a certified solar cell (PCE: 10%), as shown in Figure S10 (Supporting Information). To the best of our knowledge, this is the highest certified *V*_oc_ of PbS CQDSCs with a planar device structure of FTO/ZnO/PbS‐I/PbS‐EDT/Au, as shown in Figure 5b. In comparison to the previous certified PbS CQDSCs fabricated with various lead precursors, a prominent superiority was found in the reduced *V*_oc_ deficit (as low as 0.448 V) in our method, as shown in Table S5 (Supporting Information). To gain deep insight into this *V*_oc_ improvement, a series of measurements were performed to investigate the carrier recombination and transfer processes of the PbS CQDSCs. As shown in Figure 5c, the diode ideality factor *n* values were evaluated to be 1.37 for the Pb(acac)_2_‐PbS‐cell and 1.62 for the PbO‐PbS‐cell. The smaller *n* in the Pb(acac)_2_‐PbS‐cell implied that the trap‐assisted carrier recombination in the solar cell was effectively suppressed. Additionally, transient photovoltage (TPV) decay measurements in Figure 5d and Figure S11 (Supporting Information) showed that the electron lifetime of the Pb(acac)_2_‐PbS‐cell was much higher than that of the PbO‐PbS‐cell in test voltage range, evidencing the positive effect of the Pb(acac)_2_ on restraining the carrier recombination of solar cells. This longer electron lifetime of the Pb(acac)_2_‐PbS‐cell, consistent with the longer PL lifetime of the Pb(acac)_2_‐PbS‐I CQDs, suggested the suppression of the carrier recombination and benefited the minimized *V*_oc_ loss of the advanced photovoltaic cells.

Furthermore, the Mott–Schottky curves from the capacitance–voltage (*C*−*V*) test showed that the built‐in potential (*V*_bi_) of the Pb(acac)_2_‐PbS‐cell was 0.71 V, whereas that of the reference device was 0.63 V (Figure 5e). The Kelvin probe force microscope (KPFM) images in Figure 5f and Figure S12 (Supporting Information) showed that the surface potential difference at the ZnO/Pb(acac)_2_–PbS–I interface was much larger than that of the ZnO/PbO‐PbS‐I, further confirming the stronger built‐in field in the Pb(acac)_2_‐PbS‐cell observed from the *C*−*V* test. The larger *V*_bi_ in the Pb(acac)_2_‐PbS‐cell not only contributed to the *V*_oc_ enhancement but also accelerated the drift motion of the photo‐generated carriers. In addition, the diffusion length of the carriers was calculated to be 116.2 nm for the Pb(acac)_2_‐PbS film, which was much longer than that of the PbO‐PbS film (72.3 nm). This evidence indicated that the Pb(acac)_2_ precursor reduced the traps of the PbS CQDs by optimizing the surface ligand environment during the synthesis process. Therefore, it suppressed the trap‐related carrier recombination, enlarged the *V*_bi_ of the CQDSCs, and improved the carrier drift/diffusion transport, which induced the improvement of the *V*_oc_ and *J*_sc_ in the CQDSCs.

Previous investigations have observed the detrimental impact of OH on the photostability of CQDSCs.^[^ ^41^, ^42^ ^]^ Thus, we recorded the efficiency variation of two unencapsulated solar cells during the ambient storing time. As shown in Figure S13a (Supporting Information), the Pb(acac)_2_‐PbS‐cells maintained 95.83% of their initial PCE after ambient storing for 150 days, whereas the PbO‐PbS‐cells only maintained 87.46% of their initial PCE. Furthermore, the PCE of the unencapsulated Pb(acac)_2_‐PbS‐cells was degraded to 91.41% of the original value after 2000 min of continuous light soaking in ambient conditions. In contrast, we found that the PbO‐PbS‐cells presented fast degradation and only maintained 80.26% of their original PCE for the same condition, as shown in Figure S13b (Supporting Information). As a result, we concluded that the Pb(acac)_2_ precursor could promote the storing and light‐soaking stability of CQDSCs by reducing the surface OH ligands of the CQDs during the synthesis process.

As evidenced by the above analyses of the surface chemistry, we could safely determine the conclusion that the Pb(acac)_2_ precursor remarkably curtailed the number of OH ligands by means of avoiding water byproducts during the synthesis of PbS‐OA CQDs. This effect of Pb(acac)_2_ precursor on inhibiting OH ligands remained during the ligand exchange process and provided more reaction sites to bind iodine for the trap passivation of the PbS‐OA CQDs. Compared with the post‐synthesis surface hydroxyl inhibition strategies, accurate control of the surface ligand environment with precursor engineering in synthesis not only solved the issue of surface hydroxyl bonding from the root but also enhanced the monodispersity of CQDs for the fabrication of high‐efficiency PbS CQDSCs. Consequently, the Pb(acac)_2_ precursor suppressed the nonradiative carrier recombination, reduced energetic losses, and facilitated the carrier drift/diffusion transport, which was conducive to reducing the *V*_oc_ deficit and improving the efficiency of PbS CQDSCs. In fact, the avoidance of water byproducts during the synthesis of PbS CQDs did not completely eliminate surface hydroxylation. A small amount of OH group was still observed from XPS analysis, which was principally attributed to the H_2_O absorbed from the atmosphere during the sample preparation and characterization. Hence, the further efficiency improvement of PbS CQDSCs may be achieved through the entire elimination of OH ligands by avoiding water during synthesis in parallel with isolating water adsorption from the experimental environment.

## 3 Conclusions

In summary, we utilized a Pb(acac)_2_ precursor to optimize the surface ligand environment of PbS CQDs during the synthesis process. Compared with the conventional PbO precursor, the Pb(acac)_2_ precursor avoided water byproducts and thus reduced the number of water‐generated OH ligands during the Pb(OA)_2_ intermediate reaction process. As a result of the reduction of surface hydroxylation, a remarkable enhancement in the iodine ligand binding on PbS CQDs provided better passivation of the surface trap states, which was beneficial for suppressing the carrier nonradiative recombination and enhancing the carrier transporting in PbS CQD films. Finally, we obtained an enhanced PCE with an outstanding certified *V*_oc_ in parallel with long‐term photostability. With these benefits, we confirmed that this research will provide a new avenue for optimizing the surface chemistry of PbS CQD by means of the synthesis process for high‐performance device applications.

## Conflict of Interest

The authors declare no conflict of interest.

## Supporting information

Supporting Information

## Data Availability

The data that support the findings of this study are available in the supplementary material of this article.
