# Supplementary material for: Precursor Chemistry Enables the Surface Ligand Control of PbS Quantum Dots for Efficient Photovoltaics
Source: Adv Sci (Weinh). 2022 Nov 16;10(4):2204655. doi: 10.1002/advs.202204655 (PMC9896031; doi:10.1002/advs.202204655)
Supplement: Supplementary file 1 — Supporting Information [file ADVS-10-2204655-s001.pdf]

Supporting Information

Precursor Chemistry Enables the Surface Ligand Control of PbS Quantum Dots for Efficient Photovoltaics

Chao Wang, Yinglin Wang, Yuwen Jia, Hai Wang, Xiaofei Li, Shuai Liu, Xinlu Liu, Hongbo Zhu, Haiyu Wang, Yichun Liu and Xintong Zhang**

C. Wang, Prof. Y. Wang, Y. Jia, X. Li, S. Liu, X. Liu, H. Zhu, Prof. Y. Liu, Prof. X. Zhang

Key Laboratory of UV-Emitting Materials and Technology of Chinese Ministry of Education, Northeast Normal University, Changchun 130024, China.

E-mail: (wangyl100@nenu.edu.cn; xtzhang@nenu.edu.cn)

Dr. H. Wang, Prof. H. Wang

State Key Laboratory on Integrated Optoelectronics College of Electronic Science and Engineering Jilin University, Changchun 130012, China.

Experimental Section

Synthesis of PbS CQDs: The PbS CQDs covered with oleate (PbS-OA) were prepared by means of a widespread hot injection method according to previous literatures with moderate modification.[1] Typically, 2 mmol of $Pb(acac)_2$ or PbO and 1.85 mL OA were added into 20 mL of ODE solution in formation of transparent solution with a 120 °C heat-treatment under vacuum ambiance for 2h in a flask. Then the sulfide precursor mixture solution of 180 μL of hexamethyldisilathiane (TMS_2S) and 5 mL of ODE was rapidly injected into the reaction flask for 1 min under nitrogen-filled condition. After reaction, the heating device was removed to cool the suspension to room temperature naturally under continuous nitrogen protection. Next, acetone and hexane were applied alternately for the purification of the synthesized PbS-OA three times. Finally, the PbS-OA solid were extracted from rinsing agent by centrifugation at 6000 rpm for 5 min and redispersed in the octane with a 50 mg mL^{-1} as well as stored in the refrigerator for subsequent device fabrication.

The $Pb(OA)_2$ intermediates were prepared by cooling the reaction mixture to room temperature without the injection of sulfur source and upon purification using the same process described above for the CQDs synthesis to obtain white precipitate, then followed by overnight drying under vacuum. The white solid was stored in refrigerator at 4 °C for subsquent characterization.

The precursor solution was synthesized by cooling the transparent mixture solution to room temperature for the solution-phase FTIR measurement.

ZnO Sol-Gel Preparation: ZnO sol−gel was prepared based on a slight modification of reported method.[2] A mixture solution composed of 0.5 g of zinc acetate dihydrate, 135 μL of ethanolamine and 5 mL of 2-methoxyethanol was stirred overnight at room temperature to generate colorless and transparent gel.

PbS-I Preparation and Film Fabrication: Solution-phase ligand exchange process was used to prepare PbS-I solution following the reported method.[3] 0.4612 g PbI_2, 0.0734 g $PbBr_2$ and 0.02 g CH_3COONH_4 were dissolved in 5 mL N,N-Dimethylformamide (DMF) solution and stirred for 15 min in formation of transparent solution. Subsequently, 5 mL of PbS-OA (20 mg mL^{-1}) in octane was pipetted into the mixed solution, which was vigorously stirred for 5 min and the PbS CQDs completely transferred from octane to polar DMF phase for the purpose of removing OA ligands and forming PbS-I solution. Octane was added into polar phase to rinse PbS-I three times. Then, PbS-I solid was precipitated by centrifuge (6000 rpm for 3 min) with 2 mL toluene as antisolvent. After vacuum drying for 15 min, PbS-I solid was re-dispersed in *n*-butylamine at a concentration of 200 mg mL^{-1} for n-type ink preparation.

PbS CQDs Solar Cell Fabrication: FTO glass substrates were ultrasonically cleaned with solvents and treated with UV and oxygen plasma. Then the ZnO layer was deposited by virtue of spin-coating ZnO sol-gel solution onto FTO at 2000 rpm for 30 s and then annealed at 200 °C for 15 min under ambient environment. Subsequently, the prepared PbS-I inks were spin-cast onto the ZnO layer at 2500 rpm for 30 s to construct light absorption layer in the ambient. Next, for the PbS-EDT layer deposition, 50 μL of PbS-OA (50 mg mL^{-1}) was dropped and spin-coated on PbS-I thin films at 2500 rpm for 10 s and then treated with EDT solution (0.02 vol% in acetonitrile) and acetonitrile rinsing three times. Finally, the Au electrodes were fabricated on the top of device by thermal evaporation technique using the home-made shadow mask. The unencapsulated PbS CQDSCs in this work were constructed to test the device performance. The active area of PbS device was 0.0314 cm^{-2}.

Density of Oleate Ligand Determination: We carried out the measurements to quantify the the number of oleate ligands per unit area according to previous literatures.[4,5] First, the absorbance at 400 nm (A_{400}) was measured to caluculate the concentration (C_0) of the PbS-OA CQDs using Beer's law: $A_{400} = \varepsilon b C_0$, where b is the distance of light through the sample, ε is the molar extinction coefficient, associated with the CQDs size according to the relation

$$\varepsilon_{400} = (0.0233 \pm 0.0001) d^3 \mathrm{cm}^{-1}/\mu\mathrm{M} \tag{1}$$

Then, 2 mM ferrocene (Fc) was employed as an internal standard in ^{1}H NMR to determine ligand populations in the PbS-OA CQD samples. The total concentration of oleate species [OA] was calculated from the relative strength of the olefin proton resonance in ^{1}H NMR spectra using the equation

$$[\mathrm{OA}] = [\mathrm{Fc}] \times \frac{10}{\int \mathrm{Fc}} \times \frac{\int \mathrm{Olefin}}{2} \tag{2}$$

The $\int$ Olefin and $\int$ Fc can be obtained from the integral area at the peak of 5.33 ppm and 4.16 ppm for olefin and Fc proton resonance in ^{1}H NMR spectra. Finally, by dividing [OA] by the concentration of CQDs (C_0) as determined from the absorbance, a ratio of [OA]/C_0 could be obtained to calculate the number of oleate ligands per CQD. The density ($\sum_{OA}$) of oleate ligand (per unit area) on CQD surface could be calculated by the expression

$$\frac{[\mathrm{OA}]}{[C_0]} \div 4\pi r^2 \tag{3}$$

where r is the radius of CQD.

Material Characterization: For the preparation of samples, 10 μL of oleic acid capped PbS QDs was dissolved in 3 mL of octane and put into quartz cuvette. Film samples were prepared by spin-coating the PbS-I solution on quartz or FTO glass. UV–vis–NIR spectrophotometer (UH–4150, Shimadzu, Japan) was utilized for recording the light absorption spectra properties

of PbS CQDs solution and CQDs films. The steady-state PL spectrum was recorded using a Photoluminescence (HORIBA JOBIN YVON) equipped with a 532 nm laser as the excitation source. Time-resolved PL spectra were recorded using a Hamamatsu Photonics (Shimokanzo, Iwata, Japan) with a cathode sensitive to near-IR radiation. Nuclear magnetic resonance spectroscopy (NMR). The collected $Pb(OA)_2$ solid or pure oleic acid (20 mM) was dissolved into the deuterated chloroform ($CDCl_3$) for ^1H NMR spectra investigation based on a Bruker Avance 600 MHz instrument. The PbS-OA solid was precipitated by centrifuge (6000 rpm for 3 min) with acetone and dried under nitrogen. X-ray diffraction patterns (XRD) data were measured with Rigaku D/max-2500 X-ray diffractometer. X-ray photoelectron spectroscopy (XPS) was performed on a Kratos AXIS ULTRA X-ray photoelectron spectroscope equipped with Al Kα excitation. Surface potential distribution at the PbS-I/ZnO interface was obtained by Kelvin probe force microscope (KPFM) (ICON, Veeco/Bruker) equipped with KP-6500 Digital Kelvin Probe System. FTIR spectrum of the PbS CQD solution was measured using Agilent Technologies cary 630 in the transmittance mode. The Transmission Electron Microscopy (TEM) image of the PbS CQDs was carried out using a FEI Talos F200S at 300 KV. For TA measurement, the femtosecond transient absorption (TA) spectroscopy measurements were implemented using a regenerative amplified Ti:sapphire laser system (CPA-2010, Clark−MXR Inc.) with wavelength of 775 nm, 1 KHz repetition rate, 150 fs pulse width. In this work, the CQDs were excited by a pump light with wavelength of 640 nm. The pump light intensity was 5 μJ cm^{-2}. Samples for TA spectroscopy were spin coated PbS-I in BTA (100 mg mL^{-1}) solution on quartz substrates. Urbach energy (E_u) was utilized to evaluate the energetic disorder in PbS-I CQDs films, which was obtained by a steep slope of absorption edge through the equation

$$\alpha(E) = \alpha_0 exp\,(h\nu/E_\mathrm{u}) \tag{4}$$

where α is the absorption coefficient, $h\nu$ is the photon energy and α_0 is constant.

Device Characterization: The Current−voltage ($J-V$) characteristics of PbS CQDSCs was tested under a simulated AM 1.5 G (100 mW cm^{-2}) solar spectrum provided by Keithley 2400 source meter with a 450 W Xenon lamp (Enlitech, Model SS−F5−3A, Taiwan) and the working area of CQDSCs was 0.0314 cm^2 determined by black mask. The spectral mismatch was calibrated using a reference solar cell (Newport). The $J-V$ cuves were measure by scanning the bais forward (0-0.7 V). For photostability tests, the solar cells were light soaked at open-circuit conditions under simulated AM1.5G 100 mW cm^{-2} illumination in ambient conditions (25 °C, 30% RH). After the $J-V$ measurement, the solar cells were stored in the dring cabinet under humidity of 25% RH. The plot of External quantum efficiency datas

(EQE) were attained using a Zolix Solar Cell Measurement System equipped with a xenon lamp (QE−LD), monochromatic illumination and a light intensity detection system. Internal quantum efficiency (IQE) was obtained according to the equation

$$IQE = \frac{EQE}{1-R}, \quad (5)$$

where R is reflectivity of PbS CQDs film. Transient photovoltage (TPV) measurement was used to probe the V_{oc}-dependent charge recombination. The range voltage was set from −0.5 V to 1 V with the frequency of 1kHz and a steady-state white light was applied for "light bias". A voltage transient with fluctuation of 10 mV was measured by a light with wavelength of 635 nm as optical perturbation. Mott−Schottky curves and capacitance−voltage ($C-V$) were applied to estimate V_{bi} and calculate the carrier concentration by the equation,

$$\frac{1}{C^2} = \frac{2(V_{\mathrm{bi}}-V)}{A^2 e\varepsilon_0\varepsilon_{\mathrm{r}}N} \quad (6)$$

where V_{bi} is the built-in potential obtained by fitting the linear region of $1/C^2$, A is the area of illumination in the solar cells, N is carrier concentration, ε_0 is vacuum permittivity, ε_r is the relative dielectric constant of 18.7 for CQD solid film.[6]

The device structuration of FTO/PbS-I/Al was engineered for the space charge limiting current (SCLC) measurement. The voltage was set from 0 V−0.5 V to scan as-prepared device under dark condition and the density of trap states (N_{traps}) could be calculated according to relation,

$$N_{\mathrm{trap}} = \frac{2\varepsilon_0\varepsilon_{\mathrm{r}}V_{\mathrm{TFL}}}{eL^2} \quad (7)$$

where e, L, and V_{TFL} were the elementary charge, the thickness of CQD solid film, and trap-filled limit voltage, respectively. SCLC model with the logarithm of the $I-V$ curve in the dark, suggesting transport mechanism and trap density with a linear ohmic regime (n = 1) at low bias, a trap-filled regime (n > 3) and a trap-free Child's regime (n = 2) at high bias. Furthermore, mobility was acquired from the SCLC measurement based on equation,

$$J = \frac{9}{8}\varepsilon_0\varepsilon_{\mathrm{r}}\mu\frac{V^2}{L^3} \quad (8)$$

where μ is the charge carrier mobility.

The variation of V_{oc} along with the light intensity (I) were explored based on the function of

$$V_{\mathrm{oc}} = \frac{nkT}{e}\ln\left(\frac{J_{\mathrm{sc}}}{J_0}\right) \quad (9)$$

where n is the diode ideality factor, k is the Boltzmann constant, T is temperature, e is the elementary charge, J_0 is reverse bias saturation current density.

The diffusion length of the carriers was calculated according to the formula

$$L_{\mathrm{D}} = \sqrt{\frac{k_{\mathrm{B}}T}{e}\mu\tau} \qquad (10)$$

where k_{B} is Boltzmann constant, T is temperature in Kelvin, e is elementary charge, τ is carrier lifetime and μ is electron mobility, which was measured from aforementioned SCLC experiments.

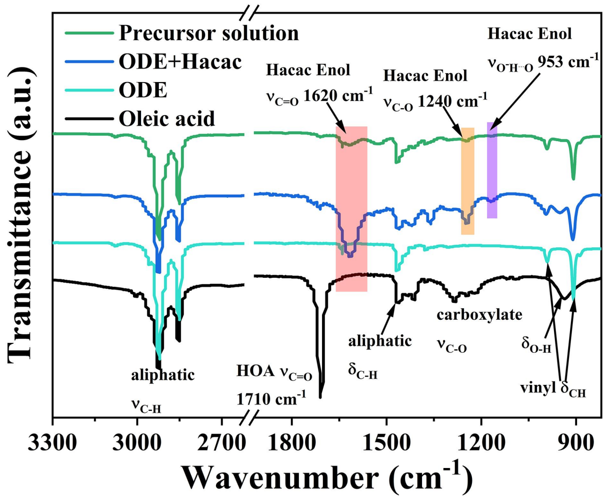

Figure S1. FTIR spectra recorded in solution-phase oleic acid, ODE, the mixture of ODE and acetylacetone (Hacac) and the synthesized precursor solution.

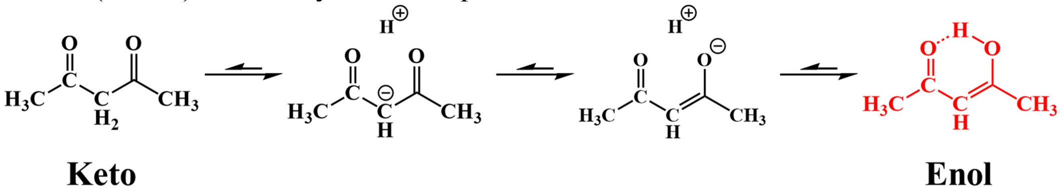

Figure S2. Keto-enol equilibrium of acetylacetone.

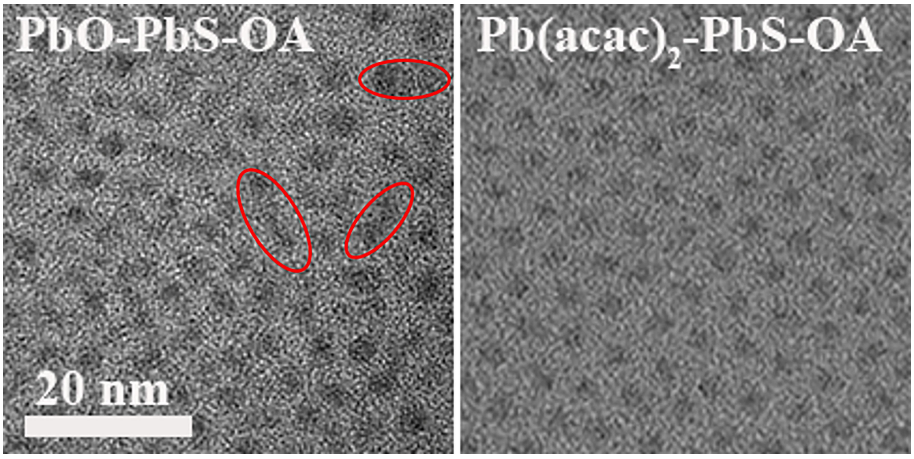

Figure S3. The TEM image of PbS-OA using PbO and Pb(acac)$_2$ precursors.

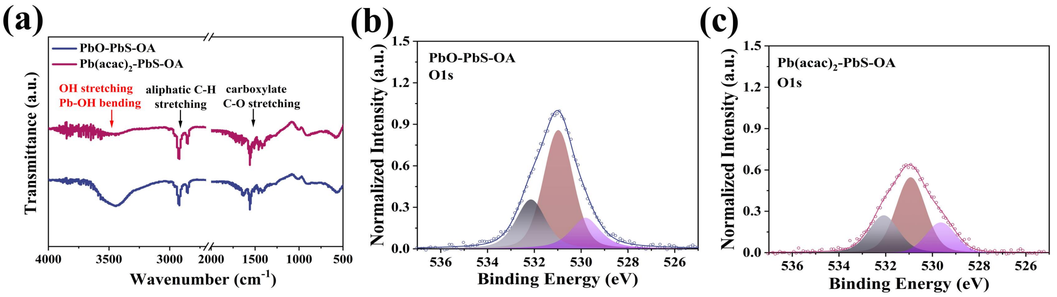

Figure S4. Characterizations of PbS-OA. (a) Fourier transform infrared (FTIR) spectra of PbO-PbS-OA and Pb(acac)$_2$-PbS-OA. O1s XPS spectra of (b) PbO-PbS-OA. (c) Pb(acac)$_2$-

PbS-OA.

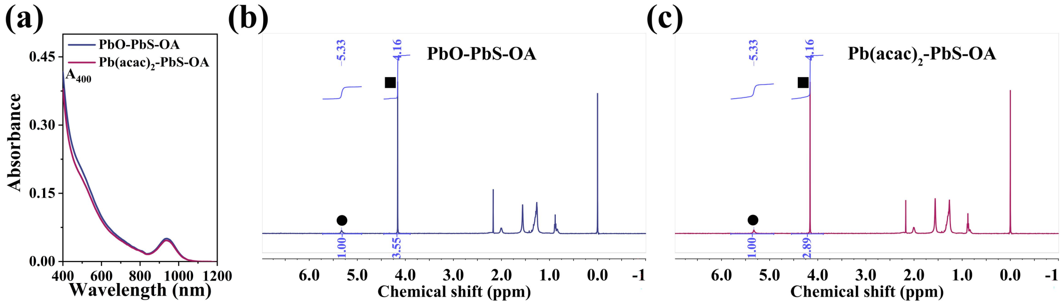

Figure S5. (a) The absorbance of PbO-PbS-OA and $Pb(acac)_2$-PbS-OA in $CDCl_3$ solution. ^1H NMR spectra of PbO-PbS-OA (b) and $Pb(acac)_2$-PbS-OA (c) in $CDCl_3$ solution. ● stands for the bound oleate ligands from olefin proton resonance. Peak at 4.16 ppm (■) is the ferrocene (Fc) internal standard.

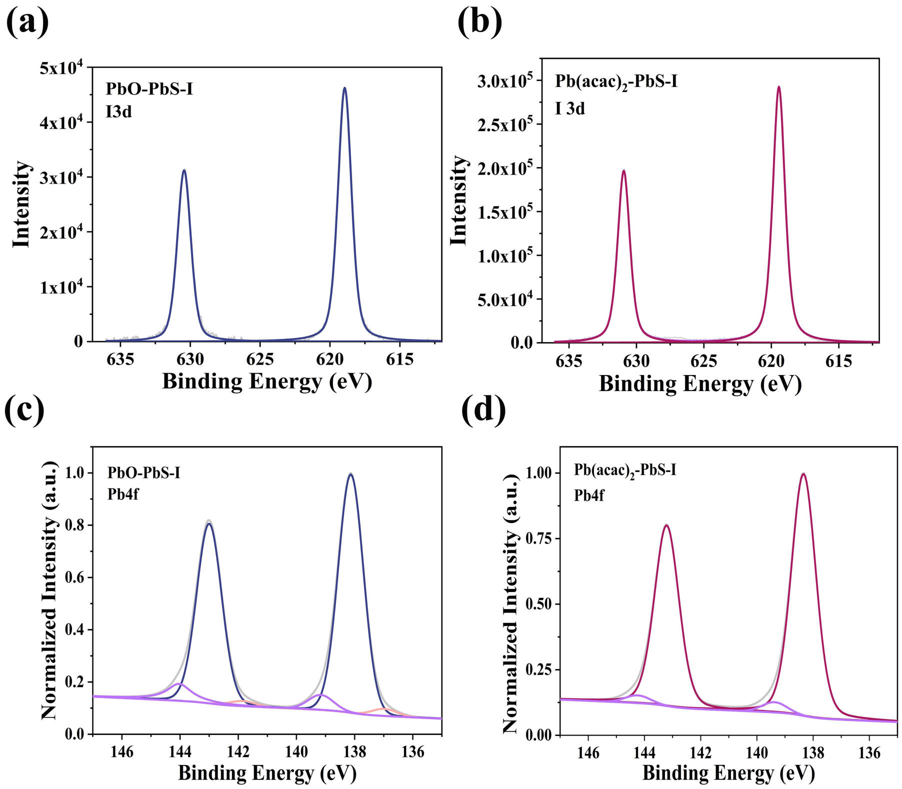

Figure S6. X-ray photoelectron (XPS) spectra of the PbS-I films. (a) I3d for PbO-PbS-I. (b) I3d for $Pb(acac)_2$-PbS-I. (c) Pb4f for PbO-PbS-I, (d) Pb4f for $Pb(acac)_2$-PbS-I.

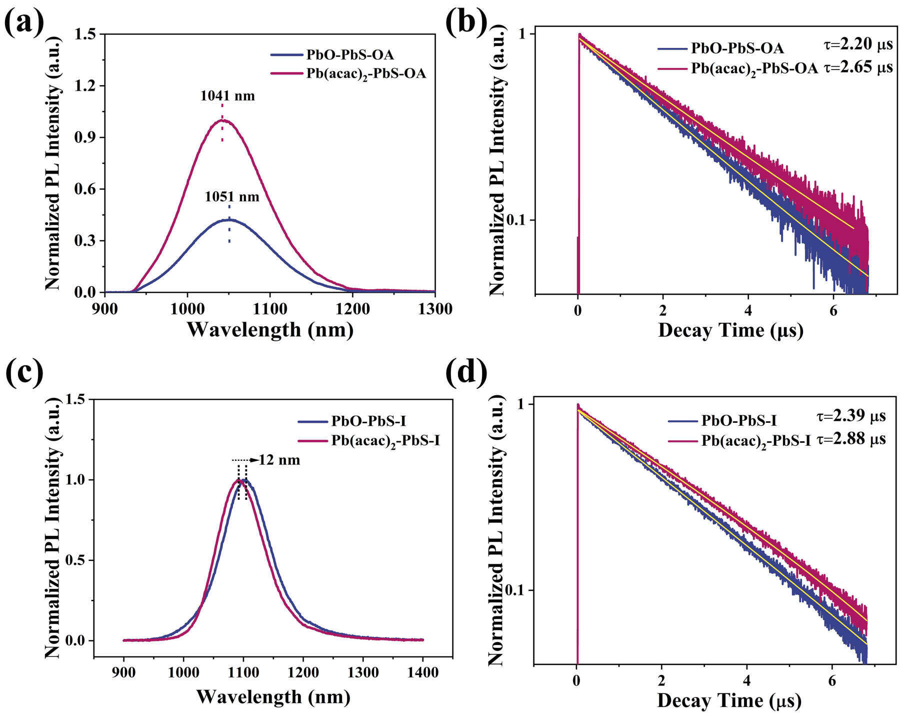

Figure S7. Photoluminescence (PL) spectra characters of PbS CQDs solution. (a) Steady-state PL spectra of PbO-PbS-OA and Pb(acac)2-PbS-OA in octane solution. The photoluminescence (PL) intensity of the Pb(acac)2-PbS-OA was much higher than that of the PbO-PbS-OA. And the Pb(acac)2-PbS-OA solution (1041nm) presented a smaller Stokes shift than that of the PbO-PbS-OA (1051nm). (b) Time-resolved PL (TRPL) measurements of the PbO-PbS-OA and Pb(acac)2-PbS-OA in octane solution. (c) The steady-state PL spectra and (d) TRPL spectra of the PbO-PbS-I and Pb(acac)2-PbS-I in DMF solution.

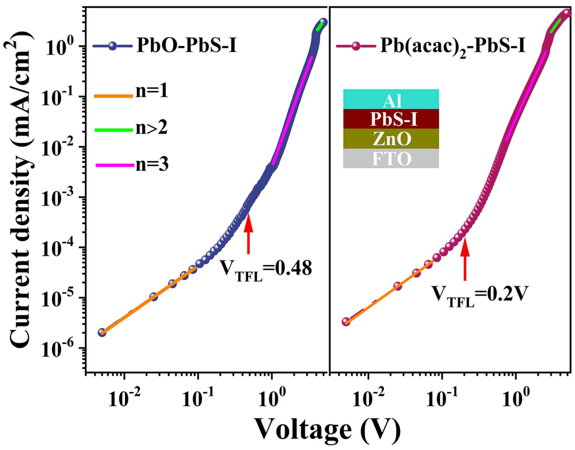

Figure S8. Space charge limited current (SCLC) panels (electron-only devices) for the PbO-PbS-I and Pb(acac)2-PbS-I devices.

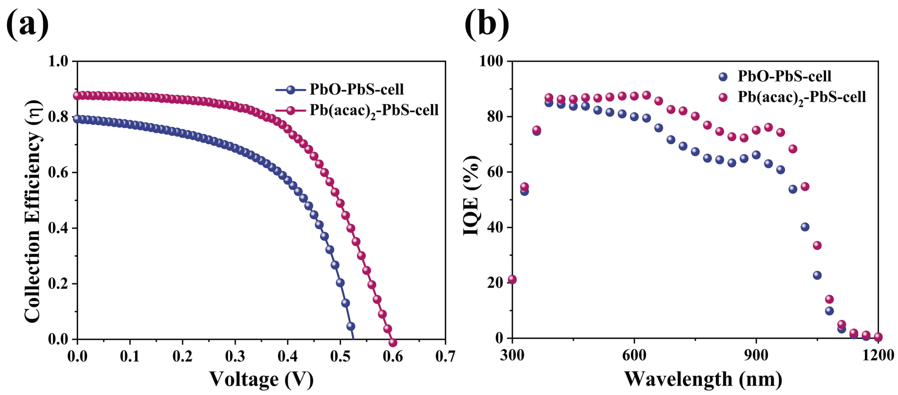

Figure S9. (a) Charge collection efficiency of the PbO-PbS-cell and $Pb(acac)_2$-PbS-cell devices. (b) Internal quantum efficiency (IQE) spectra for two devices.

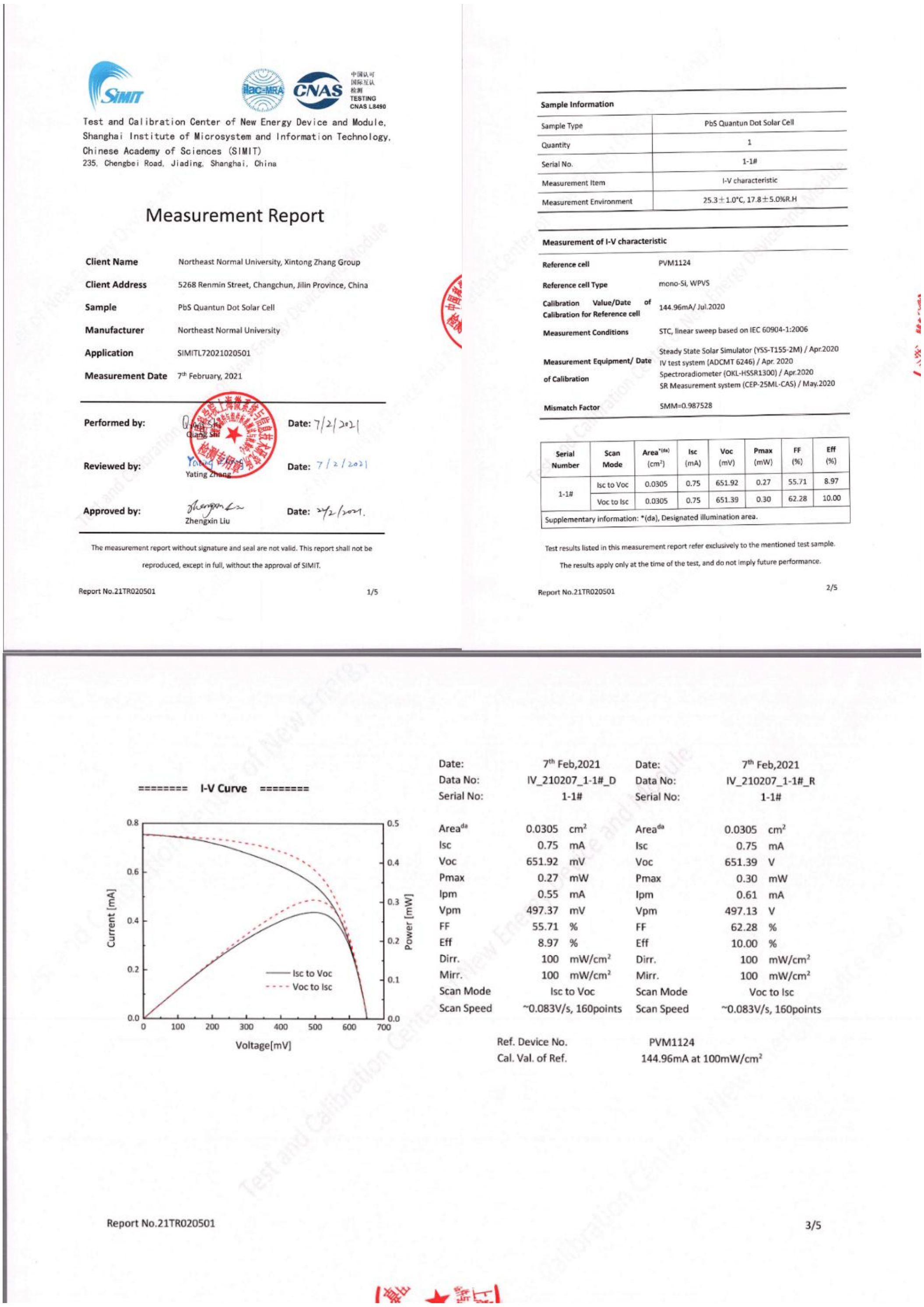

Test and Calibration Center of New Energy Device and Module, Shanghai Institute of Microsystem and Information Technology, Chinese Academy of Sciences (SIMIT)
235, Chengbei Road, Jiading, Shanghai, China

Measurement Report

Client Name	Northeast Normal University, Xintong Zhang Group
Client Address	5268 Renmin Street, Changchun, Jilin Province, China
Sample	PbS Quantun Dot Solar Cell
Manufacturer	Northeast Normal University
Application	SIMITL72021020501
Measurement Date	7th February, 2021

Performed by: Qiang Shi Date: 7/2/2021

Reviewed by: Yating Zhang Date: 7/2/2021

Approved by: Zhengxin Liu Date: 7/2/2021

The measurement report without signature and seal are not valid. This report shall not be reproduced, except in full, without the approval of SIMIT.

Report No.21TR020501 1/5

Sample Information

Sample Type	PbS Quantun Dot Solar Cell
Quantity	1
Serial No.	1-1#
Measurement Item	I-V characteristic
Measurement Environment	25.3±1.0°C, 17.8±5.0%R.H

Measurement of I-V characteristic

Reference cell	PVM1124
Reference cell Type	mono-Si, WPVS
Calibration Value/Date of Calibration for Reference cell	144.96mA/ Jul.2020
Measurement Conditions	STC, linear sweep based on IEC 60904-1:2006
Measurement Equipment/ Date of Calibration	Steady State Solar Simulator (YSS-T155-2M) / Apr.2020<br>IV test system (ADCMT 6246) / Apr. 2020<br>Spectroradiometer (OKL-HSSR1300) / Apr.2020<br>SR Measurement system (CEP-25ML-CAS) / May.2020
Mismatch Factor	SMM=0.987528

Serial Number	Scan Mode	Area*(da) (cm²)	Isc (mA)	Voc (mV)	Pmax (mW)	FF (%)	Eff (%)
1-1#	Isc to Voc	0.0305	0.75	651.92	0.27	55.71	8.97
	Voc to Isc	0.0305	0.75	651.39	0.30	62.28	10.00

Supplementary information: *(da), Designated illumination area.

Test results listed in this measurement report refer exclusively to the mentioned test sample. The results apply only at the time of the test, and do not imply future performance.

Report No.21TR020501 2/5

Date:	7th Feb,2021		Date:	7th Feb,2021	
Data No:	IV_210207_1-1#_D		Data No:	IV_210207_1-1#_R	
Serial No:	1-1#		Serial No:	1-1#	
Area^da	0.0305	cm²	Area^da	0.0305	cm²
Isc	0.75	mA	Isc	0.75	mA
Voc	651.92	mV	Voc	651.39	V
Pmax	0.27	mW	Pmax	0.30	mW
Ipm	0.55	mA	Ipm	0.61	mA
Vpm	497.37	mV	Vpm	497.13	V
FF	55.71	%	FF	62.28	%
Eff	8.97	%	Eff	10.00	%
Dirr.	100	mW/cm²	Dirr.	100	mW/cm²
Mirr.	100	mW/cm²	Mirr.	100	mW/cm²
Scan Mode	Isc to Voc		Scan Mode	Voc to Isc	
Scan Speed	~0.083V/s, 160points		Scan Speed	~0.083V/s, 160points	

Ref. Device No. PVM1124
Cal. Val. of Ref. 144.96mA at 100mW/cm²

Report No.21TR020501 3/5

Figure S10. Certified AM1.5 PCE with an excellent V_{oc} for PbS CQDSCs. This device is composed of FTO/ZnO/PbS-I/PbS-EDT/Au structure fabricated with $Pb(acac)_2$ precursor.

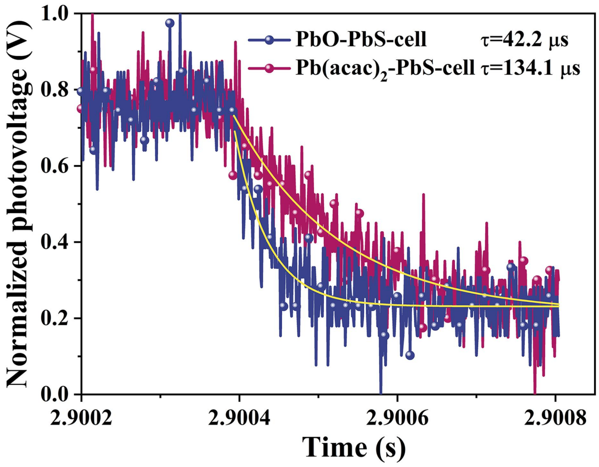

Figure S11. Transient photovoltage decay curves, the electron lifetime was calculated to be 42.2 μs and 134.1 μs for PbO-PbS-cell and $Pb(acac)_2$-PbS-cell fabricated device, respectively.

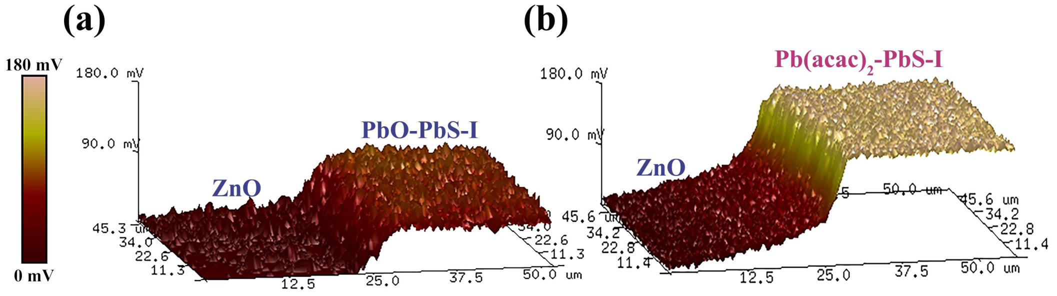

Figure S12. KPFM profiles between the ZnO and the PbS-I film interface based on different Pb precursors.

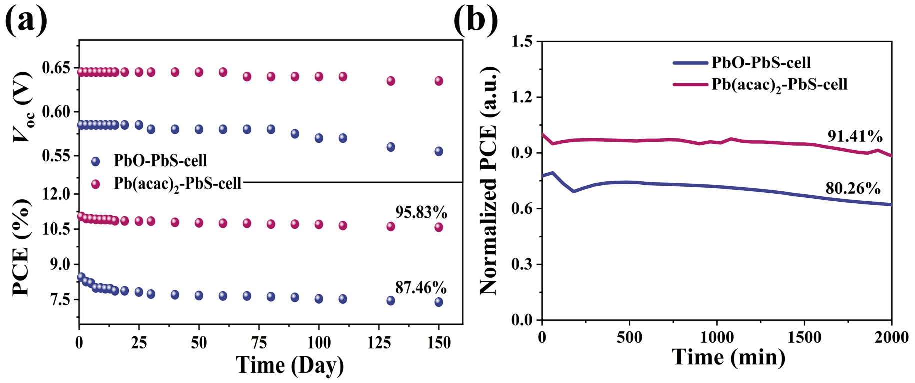

Figure S13. Device stability measurements. (a) Evolution of V_{oc} and PCE under the ambient for 150 days and (b) continuous light soaking for 2000 min.

Table S1. Summary of the deconvoluted peaks of the O1s spectra for the oleate capped PbS CQDs in Figure S1b and Figure S1c.

PbS-OA	O1s			
	Component	Peak (eV)	FWHM (eV)	Area (%)
PbO	Pb−OH	531.0	1.5	56.8
	Pb−O	529.6	1.5	17.6
	COO, CO_2	532.1	1.5	25.6
$Pb(acac)_2$	Pb−OH	531.0	1.5	52.5
	Pb−O	529.6	1.5	20.0
	COO, CO_2	532.1	1.5	27.5

Table S2. The concentration and surface ligand density for oleate capped PbS CQDs.

Precursor	Diameter (nm)	A_{400}[a)]	C_0[b)] (mM)	OA/C_0[c)]	$\sum_{OA}$ (nm^{-2})
PbO-PbS-OA	3.0	0.415	0.0198	142.42	5.04
$Pb(acac)_2$-PbS-OA	3.0	0.377	0.0180	192.22	6.80

[a)]The absorbance of PbS-OA CQDs at 400 nm; [b)]The concentration of PbS-OA CQDs; [c)]the number of oleate ligands per CQD.

Table S3. Summary of the deconvoluted peaks of the O1s spectra after ligand exchange PbS CQDs in Figure 3a.

PbS-I	O1s			
	Component	Peak (eV)	FWHM (eV)	Area (%)
PbO	Pb−OH	531.54	1.28	69.44
	Pb−O	529.77	1.1	7.56
	COO, CO_2	532.40	1.1	16.56
	OH	533.52	1.1	6.44
$Pb(acac)_2$	Pb−OH	531.53	1.2	31.38
	Pb−O	529.68	1.05	15.28
	COO, CO_2	532.39	1.05	27.55
	OH	533.48	1.05	25.79

Table S4. Summary of the deconvoluted peaks of the Pb4f spectra after ligand exchange PbS CQDs in Figure S3c and Figure. S3d.

PbS-I	Pb4f			
	Component	Peak(eV) (4f5/2)/(4f7/2)	FWHM (eV)	Area (%)
PbO	Pb−S	143.0/138.13	1.02/1.02	88.9
	COO:Pb	144.04/139.17	1.02/1.02	8.5
	Metallic Pb	141.84/136.97	1.02/1.02	2.6
$Pb(acac)_2$	Pb−S	143.21/138.34	1.02/1.02	96.3
	COO:Pb	144.24/139.37	1.02/1.02	3.7

Table S5. Analysis of V_{oc} loss for certified AM 1.5 PbS CQDSCs with various Pb precursors based on similar device structure.

Pb precursor	Device structure	PCE (%)	V_{oc} (V)	E_g^{onset} (eV)	$V_{oc,loss}$ (V)	Ref.
$Pb(Ac)_2·3H_2O$	ITO/ZnO/PbS-TBAI/PbS-EDT/Au	8.55	0.555	1.13	0.575	[7]
PbO	ITO/ZnO/PbS-MAI/PbS-EDT/Au	10.6	0.610	1.11	0.500	[8]
PbO	ITO/ZnO/PbS-I/PbS-EDT/Au	11.28	0.611	1.08	0.469	[9]
$Pb(Ac)_2·3H_2O$	ITO/ZnO/PbS-I/PbS-EDT/Au	10.62	0.620	1.15	0.530	[10]
PbO	ITO/ZnO/PbS-I/PbS-EDT/Au	12.01	0.647	1.11	0.463	[11]
$Pb(acac)_2$	FTO/ZnO/PbS-I/PbS-EDT/Au	10.00	0.652	1.10	0.448	This work

Note that the $V_{oc,loss}$ was calculated according to equation $V_{oc,\ loss} = \frac{E_g^{onset}}{e} - V_{oc}$, reported by Sargent group and Bawendi group[12,13]. E_g^{onset} was confirmed by absorption/external quantum efficiency onset.

Reference

[1] M. A. Hines and G. D. Scholes, *Adv. Mater*. **2003**, 15, 1844.

[2] M. Liu, F. P. de Arquer, Y. Li, X. Lan, G. H. Kim, O. Voznyy, L. K. Jagadamma, A. S. Abbas, S. Hoogland, Z. Lu, J. Y. Kim, A. Amassian and E. H. Sargent, *Adv. Mater*. **2016**, 28, 4142.

[3] J. Chen, S. Zheng, D. Jia, W. Liu, A. Andruszkiewicz, C. Qin, M. Yu, J. Liu, E. M. J. Johansson and X. Zhang, *ACS Energy Lett*. **2021**, 6, 1970.

[4] I. Moreels, K. Lambert, D. Smeets, D. Muynck, T. Nollet, J. C. Martins, F. Vanhaecke, A. Vantomme, C. Delerue, G. Allan, and Z. Hens, *ACS Nano* **2009**, 3, 3023.

[5] A. Roberge, J. Dunlap, F. Ahmed, and A. Greytak, *Chem. Mater*. **2020**, 32, 6588.

[6] M. Gu, Y. Wang, F. Yang, K. Lu, Y. Xue, T. Wu, H. Fang, S. Zhou, Y. Zhang, X. Ling, Y. Xu, F. Li, J. Yuan, M. A. Loi, Z. Liu and W. Ma, *J. Mater. Chem. A* **2019**, 7, 15951.

[7] C. H. Chuang, P. R. Brown, V. Bulovic and M. G. Bawendi, *Nat. Mater*. **2014**, 13, 796.

[8] X. Lan, O. Voznyy, F. P. Garcia de Arquer, M. Liu, J. Xu, A. H. Proppe, G. Walters, F. Fan, H. Tan, M. Liu, Z. Yang, S. Hoogland and E. H. Sargent, *Nano Lett*. **2016**, 16, 4630.

[9] M. Liu, O. Voznyy, R. Sabatini, F. P. García de Arquer, R. Munir, Ahmed H. Balawi, X. Lan, F. Fan, G. Walters, Ahmad R. Kirmani, S. Hoogland, F. Laquai, A. Amassian and Edward H. Sargent, *Nat. Mater*. **2017**, 16, 258.

[10] Y. Wang, K. Lu, L. Han, Z. Liu, G. Shi, H. Fang, S. Chen, T. Wu, F. Yang, M. Gu, S. Zhou, X. Ling, X. Tang, J. Zheng, M. A. Loi and W. Ma, *Adv. Mater*. **2018**, 30, 1704871.

[11] J. Xu, O. Voznyy, M. Liu, A. R. Kirmani, G. Walters, R. Munir, M. Abdelsamie, A. H. Proppe, A. Sarkar, F. P. Garcia de Arquer, M. Wei, B. Sun, M. Liu, O. Ouellette, R. Quintero-Bermudez, J. Li, J. Fan, L. Quan, P. Todorovic, H. Tan, S. Hoogland, S. O. Kelley, M. Stefik, A. Amassian and E. H. Sargent, *Nat. Nanotechnol*. **2018**, 13, 456.

[12] J. Jo, Y. Kim, J. Choi, F. P. Arquer, G. Walters, B. Sun, O. Ouellette, J. Kim, A. H. Proppe, R. Quintero-Bermudez, J. Fan, J. Xu, C. Tan, O. Voznyy, and E. H. Sargent, *Adv. Mater*. **2017**, 29, 1703627.

[13] C. H. Chuang, A. Maurano, R. E. Brandt, G. W. Hwang, J. Jean, T. Buonassisi, V. Bulovic and M. G. Bawendi, *Nano Lett* **2015**, 15, 3286.
